# Innovative Application of Chatbots in Clinical Nutrition Education: The E+DIEting_Lab Experience in University Students

**DOI:** 10.3390/nu18020257

**Published:** 2026-01-14

**Authors:** Iñaki Elío, Kilian Tutusaus, Imanol Eguren-García, Álvaro Lasarte-García, Arturo Ortega-Mansilla, Thomas A. Prola, Sandra Sumalla-Cano

**Affiliations:** 1 Research Group on Foods, Nutritional Biochemistry and Health, European University of the Atlantic, 39011 Santander, Spain; imanol.eguren@uneatlantico.es (I.E.-G.); alvaro.lasarte@alumnos.uneatlantico.es (Á.L.-G.); 2 Faculty of Health Sciences, University of Cuanza, Cuito 250, Bié, Angola; 3 Faculty of Health Sciences, University of La Romana, La Romana 22000, Dominican Republic; 4 Higher Polytechnic School, European University of the Atlantic, 39011 Santander, Spain; kilian.tutusaus@uneatlantico.es (K.T.); arturo.ortega@uneatlantico.es (A.O.-M.); 5 Higher Polytechnic School, Nutrition and Sport, Universidad Internacional Iberoamericana, Campeche 24560, Mexico; 6 Faculty of Social Sciences and Humanities, European University of the Atlantic, 39011 Santander, Spain; thomas.prola@uneatlantico.es; 7 Faculty of Health Sciences, Universidad Internacional Iberoamericana, Arecibo, PR 00613, USA; 8 Department of Health, Nutrition and Sport, Universidad Internacional Iberoamericana, Campeche 24560, Mexico

**Keywords:** chatbots, clinical nutrition education, dietetic students, artificial intelligence

## Abstract

**Background/Objectives**: The growing integration of Artificial Intelligence (AI) and chatbots in health professional education offers innovative methods to enhance learning and clinical preparedness. This study aimed to evaluate the educational impact and perceptions in university students of Human Nutrition and Dietetics, regarding the utility, usability, and design of the E+DIEting_Lab chatbot platform when implemented in clinical nutrition training. **Methods**: The platform was piloted from December 2023 to April 2025 involving 475 students from multiple European universities. While all 475 students completed the initial survey, 305 finished the follow-up evaluation, representing a 36% attrition rate. Participants completed surveys before and after interacting with the chatbots, assessing prior experience, knowledge, skills, and attitudes. Data were analyzed using descriptive statistics and independent samples *t*-tests to compare pre- and post-intervention perceptions. **Results**: A total of 475 university students completed the initial survey and 305 the final evaluation. Most university students were females (75.4%), with representation from six languages and diverse institutions. Students reported clear perceived learning gains: 79.7% reported updated practical skills in clinical dietetics and communication were improved, 90% felt that new digital tools improved classroom practice, and 73.9% reported enhanced interpersonal skills. Self-rated competence in using chatbots as learning tools increased significantly, with mean knowledge scores rising from 2.32 to 2.66 and skills from 2.39 to 2.79 on a 0–5 Likert scale (*p* < 0.001 for both). Perceived effectiveness and usefulness of chatbots as self-learning tools remained positive but showed a small decline after use (effectiveness from 3.63 to 3.42; usefulness from 3.63 to 3.45), suggesting that hands-on experience refined, but did not diminish, students’ overall favorable views of the platform. **Conclusions**: The implementation and pilot evaluation of the E+DIEting_Lab self-learning virtual patient chatbot platform demonstrate that structured digital simulation tools can significantly improve perceived clinical nutrition competences. These findings support chatbot adoption in dietetics curricula and inform future digital education innovations.

## 1. Introduction

The rapid evolution of digital technology is reshaping healthcare, with artificial intelligence (AI) now playing a central role in both clinical practice and health education. Among AI driven solutions, chatbots, also known as conversational agents, have gained traction for their ability to interact with users through text or voice, providing immediate access to information, guidance, and support across a range of health contexts [1,2].

Initially limited to simple administrative tasks, recent advances in Natural Language Processing (NLP) and Machine Learning (ML) have catalyzed a transition toward more sophisticated, personalized services in clinical nutrition, such as automated dietary assessment and clinical decision support. This shift represents more than a functional upgrade; it signifies a move from deterministic, rule-based systems to probabilistic models capable of handling the inherent ambiguity of human language. While these technologies offer the potential to alleviate the global burden of non-communicable diseases through scalable, real-time guidance, their integration into clinical practice presents significant challenges. The key lies in balancing conversational fluidity with the rigorous accuracy required for nutritional assessment. Consequently, analyzing how these agents interpret user intent and manage complex clinical data is essential to ensure they serve as reliable adjuncts to professional dietetic expertise [3].

Previous studies with university students in medicine, nursing, and allied health professions have shown that virtual patients and chatbots can improve perceived clinical reasoning and communication skills, although most rely on subjective outcomes and small samples [4,5,6,7]. Evidence specifically in nutrition and dietetics university students remains scarce, highlighting the need for multicentre evaluations tailored to dietetics curricula.

The growing burden of non-communicable diseases such as obesity, diabetes, and cardiovascular disorders highlights the urgent need for innovative strategies in prevention, management, and professional training [8]. Chatbots are emerging as valuable tools for addressing these challenges, offering scalable and accessible solutions for both patients and healthcare providers. In clinical nutrition, they can facilitate risk screening, support personalized dietary planning, and promote healthy behaviors, while also serving as educational aids for students and practitioners [9,10,11].

In parallel, the integration of virtual patients and digital simulations into health professions education is transforming traditional teaching methods. These tools provide interactive, realistic scenarios that help students develop clinical reasoning and decision-making skills in a safe controlled environment. Early research suggests that virtual patient simulations can enhance learning outcomes and better prepare students for real-world encounters [4,5,6].

**Figure 1:**
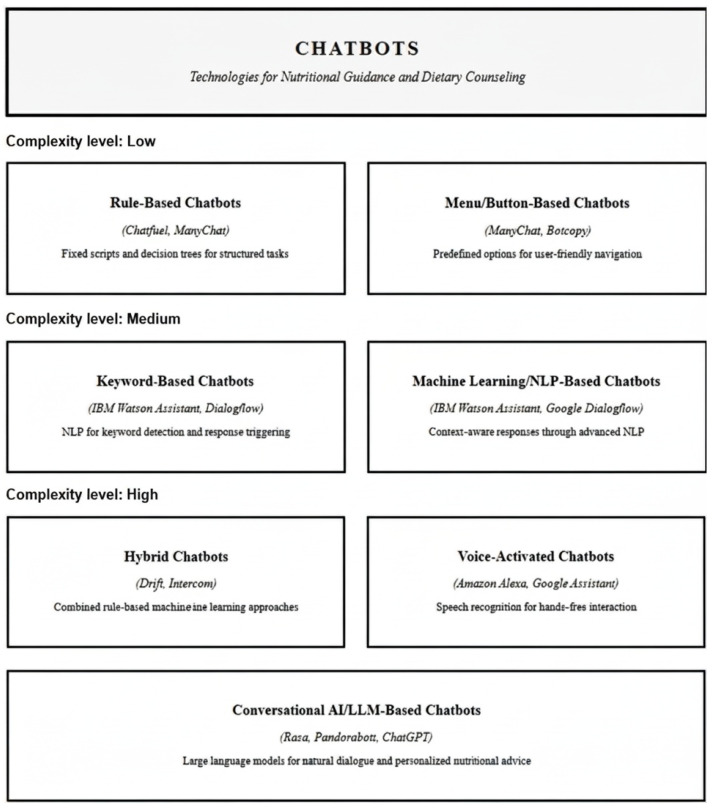
Classification of Chatbot Technologies for Nutritional Applications.

Chatbots used in healthcare and clinical nutrition can be categorized according to their underlying technology and interaction style, each offering distinct advantages and limitations [1,3,9] Figure 1.

- **Rule-Based chatbots** (e.g., Chatfuel, ManyChat): Operate using fixed scripts and decisions trees, ideal for structured, repetitive tasks like appointments scheduling or standardized information delivery.
- **Menu/button-based chatbots** (e.g., ManyChat, Botcopy): Guide users through a series of predefined options or menus, making them highly user-friendly for basic data collection and navigation.
- **Keyword-based chatbots** (e.g., IBM Watson Assistant, Dialogflow): Use Natural Language Processing (NLP) to detect keywords or phrases in user input, triggering specific responses. These offer more flexibility than rule-based bots are limited in handling complex queries.
- **Machine learning/NLP-based chatbots** (e.g., IBM Watson Assistant, Google Dialogflow): Employ advanced NLP and machine learning to interpret user intent and context, enabling dynamic, context-aware responses for complex interactions.
- **Hybrid chatbots** (e.g., Drift, Intercom): Combine rule-based logic with machine learning, balancing reliability for routine tasks and adaptability for nuanced conversations.
- **Voice-activated chatbots** (e.g., Amazon Alexa, Google Assistant): Use speech recognition and synthesis to enable hands-free, verbal interaction, enhancing accessibility.
- **Conversational AI/LL-M-based chatbots** (e.g., Rasa, Pandorabots, ChatGPT): Leverage deep learning and large language models to deliver natural, human-like dialog and tailored health advice, supporting complex decision-making and education.

While the use of AI-driven chatbots has been previously explored in other university populations, such as medical and nursing students, to enhance diagnostic skills and patient interaction [7,12,13], there is a notable lack of research specifically focused on clinical nutrition education for Human Nutrition and Dietetics students. To date, most studies in the field of dietetics have prioritized patient-oriented tools for chronic disease management rather than pedagogical resources, particularly multicentre, multilingual virtual patient chatbots aligned with Nutrition Care Process (NCP) and International Dietetics Nutrition Terminology (IDNT), for undergraduate training. This study addresses this critical literature gap by being one of the first to evaluate the integration of the E+DIEting_Lab specialized chatbot platform across multiple European universities into the clinical nutrition curriculum, providing a novel perspective on its perceived effectiveness in bridging the gap between theoretical knowledge and professional dietetic practice.

The E+DIEting_Lab platform combines multilingual, case-based virtual patients with robust security and GDPR-compliant data management, providing a structured, AI-driven environment for practicing clinical nutrition skills.

## 2. Materials and Methods

This study was conducted as part of the “Digital Lab for Education in Dietetics combining Experiential Learning and Community Service” (n° 2021-1-ES01-KA220-HED-000032074), an Erasmus+ initiative aimed at enhancing clinical nutrition education through the development and implementation of digital tools, including chatbots and virtual patients’ simulations. The project involved a multidisciplinary team of dietitians, educators, and technology specialists from several European universities and was piloted across multiple higher education institutions in Austria, Belgium, Poland, Portugal, and Spain, and was implemented between 2021 and 2024.

The study was conducted in accordance with the Declaration of Helsinki, and approved by the Institutional Review Board (Ethics Committee) of Jan Kochanowski University in Kielce (protocol code 2021-1-ES01-KA220-HED-000032074, approval date: (25 September 2023).

### 2.1. Technological Development Process

The development of the E+DIEting_Lab platform began with a comprehensive review of existing digital solutions for clinical nutrition education. The project team systematically analyzed a range of chatbot frameworks, virtual patient simulators, and educational technologies, assessing each for language processing capabilities, adaptability to clinical scenarios, multilingual support, and compatibility with learning management systems. Key criteria included usability, scalability, data security, and alignment with the NCP [14] and IDNT [15].

Based on this assessment Dialogflow (Google LLC, MountainView, CA, USA) was selected as the core technology for chatbot development due to its advanced natural language processing, multi-language support, and robust cloud infrastructure. The backend was implemented using Python 3.13.5 (Python Software Foundation, Wilmington, DE, USA) and Anaconda (Anaconda Inc., Austin, TX, USA) while cloud services such as Amazon Web Services (Amazon Web Services Inc., Seattle, WA, USA) (AWS), Microsoft Azure (Microsoft Corporation, Redmond, WA, USA) (Azure), and Google Cloud Platform (Google LLC, Mountain View, CA, USA) ensured hosting and scalability. The platform was designed for seamless integration with academic learning management systems through xAPI/cmi5 standards.

### 2.2. Platform Architecture and Security

The E+DIEting Lab self-learning platform is a robust, web-based application accessible at https://virtual-patient.edietinglab.eu/ (accessed on 12 December 2025). It offers a secure, user-friendly environment where registered users can interact with virtual patient chatbots designed to simulate diverse clinical nutrition scenarios. User registration and authentication mechanisms were implemented to ensure controlled access, supporting role differentiation among students, educators, and other stakeholders.

The platform delivers multilingual support, accommodating English, Spanish, Dutch, Portuguese, Polish, and German to maximize inclusivity and accessibility across the participating European institutions. Interaction is facilitated through the Botcopy (Botcopy Inc., Los Angeles, CA, USA), which enables both text-based and voice-enabled communication, enhancing usability and mirroring real-world conversational dynamics. This dual-modality interaction promotes engagement and replicates clinical consultation workflows more realistically.

From a technical perspective, the platform architecture embraces a cloud-native approach hosted on Google Cloud Platform, with supplementary use of Amazon Web Services (AWS) and Microsoft Azure to guarantee scalability, high availability, and fault tolerance. The backend infrastructure is developed primarily using Python 3.13.5 and Anaconda 2025.06-1, optimized for seamless integration with Dialogflow’s natural language processing engine.

Furthermore, the platform complies rigorously with the European Union’s General Data Protection Regulation (GDPR), implementing data minimization, purpose limitation, and user consent management frameworks. Anonymization and pseudonymization techniques are applied to user data sets employed for analytic and research purposes, emphasizing privacy preservation.

### 2.3. Educational Content and Scenario Design

**Figure 2:**
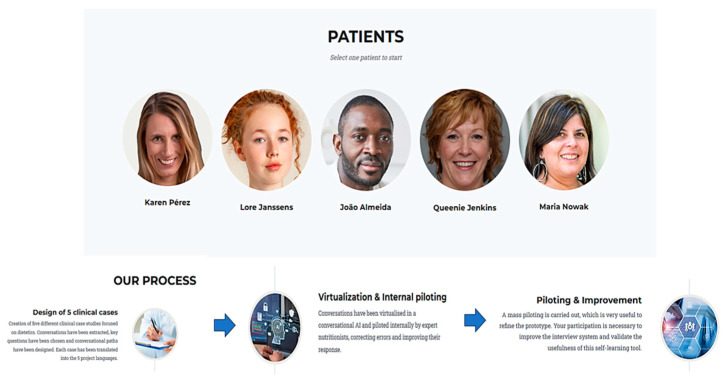
Five virtual patients created for the project E+DIEting_Lab.

A needs assessment was conducted with input from academic staff, students, and practicing professionals to identify gaps in digital competences and practical training. Five virtual patients’ scenarios were created, each based on real clinical history, anthropometric and biochemical data, dietary assessment, and interactive chatbot dialog for simulated anamnesis. It was decided that each chatbot would focus on a prevalent pathology, ultimately selecting gastroenterology, type 1 diabetes, cardiovascular disease and diabetes, obesity, and renal disease as the clinical areas of focus. Each case was paired with self-assessment questions based on IDNT to reinforce learning, Figure 2.

**Figure 3:**
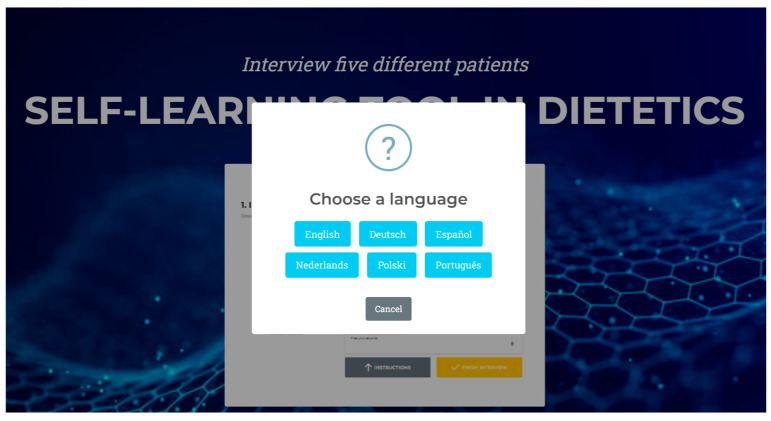
Virtual patients in English, German, Spanish, Dutch, Polish and Portuguese.

The educational content was designed to be accessible in six languages, ensuring inclusivity. The interface supports intuitive navigation, clear instructions, and allows users to download transcripts of their interactions and self-assessment results. Usability and user experience were prioritized, with interactive feedback from students and educators guiding refinements, Figure 3.

### 2.4. Platform Process Interaction

**Figure 4:**
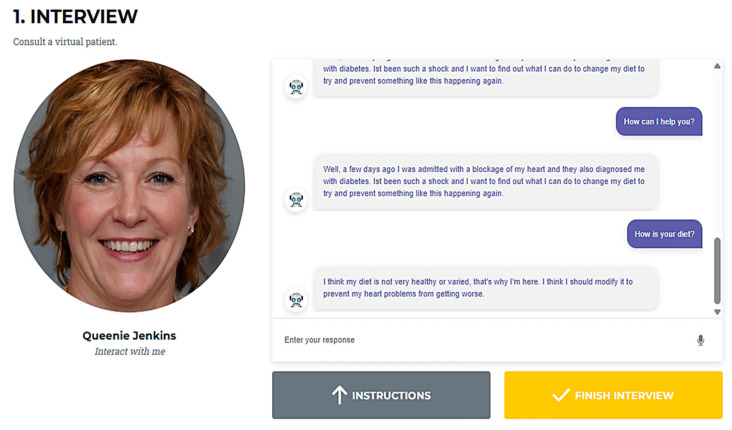
Interaction with the virtual patient Queenie Jenkins.

**Figure 5:**
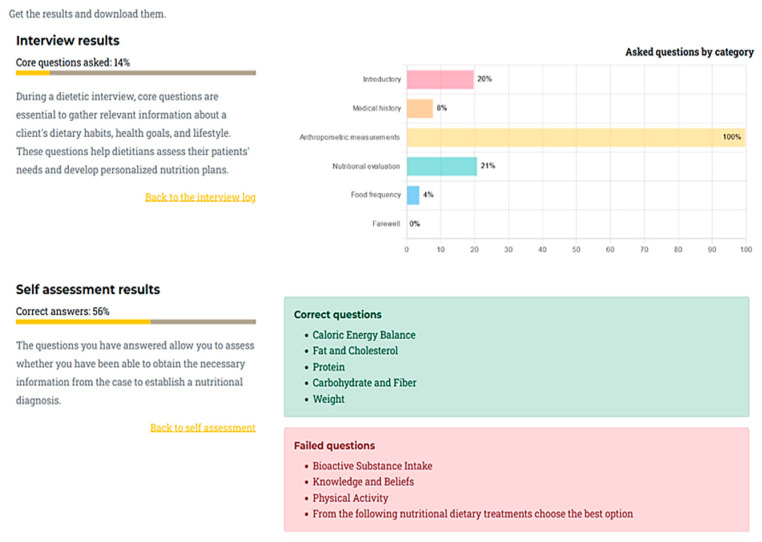
Personalize feedback after the virtual patient interaction.

The platform interaction was structured to simulate a real clinical consultation and self-reflection workflow, Figure 4 and Figure 5:

- User Registration and Access: Learners register and log in, selecting their preferred language.
- Initial Survey: Before starting, users complete a brief survey regarding previous experience with chatbots and expectations for the session.
- The user selects the virtual patient/chatbot with whom he/she wants to interact.
- Chatbot interview: Learners conduct an interactive interview with the virtual patient, posing questions via text or voice. The chatbot provides responses based on a standardized case template and prompts rephrasing if a question cannot be answered.
- Self-Assessment: After the interview, users complete a set of multiple-choice questions related to the case, based on the IDNT, covering Intake, Clinical, Behavioral and Environmental, and Dietary treatments.
- Results: The platform provides feedback on both the interview and self-assessment, highlighting strengths and areas for improvement. Users can download a summary of their interactions and results for review or document.
- Final Survey and Results: Users complete a final survey on chatbot usability and effectiveness.

This process enables students to practice clinical interviewing, apply critical thinking, and receive immediate feedback, supporting experimental learning in a safe, controlled environment.

### 2.5. Validation and Pilot Implementation

Participants were undergraduate students enrolled in Bachelor’s degree programs in Human Nutrition and Dietetics at six European universities (Austria, Belgium, Poland, Portugal, Spain), representing diverse academic years and clinical nutrition coursework.

The validation and evaluation process was structured to ensure both technical robustness and educational effectiveness. The platform underwent expert review by panels of dietitians, educators, and technologists, who assessed the clinical accuracy, usability, and pedagogical value of the tool. Data collection included:

- Performance on self-assessment quizzes, automatically scored and linked to IDNT domains.
- User satisfaction and usability feedback, gathered through structured surveys administered before and after the chatbot experience.
- Immediate pre-post evaluation of virtual patient interactions without control group.

Quantitative data were analyzed using descriptive statistics to identify trends in usage and learning outcomes, while qualitative feedback was thematically analyzed to inform iterative improvements to the platform. During the pilot process, the questions that had no answers in the proposed cases were collected and implemented into the case structure in real time to improve their usability. The evaluation emphasized effectiveness, efficiency, and user satisfaction, following established frameworks for digital health education tools.

### 2.6. Statistical Analysis

Quantitative data from pre- and post-intervention surveys were analyzed using jamovi software (version 2.3.28; The jamovi project, 2023), an open-source statistical platform built on R.

Descriptive statistics included frequencies, percentages, means, and standard deviations (SD) for categorical (sex, language, prior experience, post-intervention perceptions) and continuous Likert-scale variables (0–5 scale: 0 = very bad/not at all effective/useful; 5 = very good/highly effective/extremely useful).

Subgroup analyses by gender and nationality/language were not performed due to the exploratory nature of this pilot study and primary focus on pre-post changes across the total cohort. Descriptive demographic data are presented in Table 1.

Due to 36% attrition (475 initial → 305 final completers), independent samples *t*-tests compared separate pre-survey (*n* = 475) and post-survey (*n* = 305) samples rather than paired analyses of matched individuals. No participant matching was possible as completer IDs were not linked across surveys. Normality was assessed via Shapiro–Wilk tests and homogeneity via Levene’s test; effect sizes calculated using Cohen’s d. This approach acknowledges sample non-independence while providing valid between-group pre-post comparisons for this exploratory pilot.

Nonparametric Wilcoxon signed-rank tests supplemented ordinal analyses where parametric assumptions were violated. Statistical significance was set at *p* < 0.05 (two-tailed), without multiple comparison adjustments due to confirmatory primary outcomes.

## 3. Results

This section outlines the outcomes observed during the pilot implementation of the E+DIEting_Lab digital tools across multiple European academic institutions.

### 3.1. Sample and Participation

Between December 2023 and April 2025, the pilot enrolled 475 participants for the initial survey and 305 for the final survey, with response rates of 60.9% and 39.1%, respectively.

### 3.2. Demographic Characteristics

The sample comprised 75.4% female (*n* = 358) and 24.6% men (*n* = 117) students. Spanish speakers were predominant (41.7%, *n* = 198), followed by Polish (20.8%, *n* = 99) and Portuguese (16.6%, *n* = 79). Full distribution shown in Table 1. No subgroup analyses by gender or nationality were conducted in this pilot evaluation to maintain focus on overall platform acceptability across diverse European programs in Human Nutrition and Dietetics.

### 3.3. Educational Utility and Experience

Prior use of chatbot was reported by 43.4% of the participants (Table 2). Most participants, after the interaction with the chatbots, agreed that their practical skills related to clinical dietetics or communication were update (79.7%), and 90% acknowledged improvements in classroom practice from new training tools. Over 70% reported enhanced interpersonal skills, Table 3.

Self-rated knowledge about chatbot use in dietetics learning improved modestly but significantly from 2.32 ± 1.13 pre-survey to 2.66 ± 1.15 post survey (*p* < 0.001), representing a small medium effect. Similarly, self-assessed skills increased from 2.39 ± 1.11 to 2.79 ± 1.11 (*p* < 0.001). However, perceived effectiveness declined slightly from 3.63 ± 0.905 to 3.42 ± 1.07 (*p* = 0.002), and usefulness decreased from 3.63 ± 0.920 to 3.45 ± 1.09 (*p* = 0.018), remaining positive overall (>3.4/5). Full comparative results shown in Table 4. These patterns suggest growing familiarity tempered by realistic post-use expectations of the platform’s intent-based limitations.

Independent *t*-tests compare distinct samples (pre: *n* = 475; post: *n* = 305) due to unmatched attrition, not within-subject changes. Significant attrition (36%, 475 → 305) raises nonresponse bias concerns. Students with more favorable platform experiences may have been more motivated to complete the final survey, potentially overestimating perceived benefits. Characteristics of completers vs. non-completers were unavailable, precluding bias assessment.

## 4. Discussion

The E+DIEting_Lab platform has been used with students of the bachelor’s degree in human nutrition and Dietetics at universities in Austria, Portugal, Spain, Belgium, and Poland in various subjects throughout the program. As with all new tools, they should be included in the curriculum alongside the existing tools [16]. Students access the platform after receiving training on different pathologies and on how to carry out the nutritional care process with patients. The platform allows students to practice their skills in organizing the process of a nutritional interview and their ability to collect relevant information to establish a nutritional diagnosis and subsequently carry out the nutritional intervention. Before and after using the chatbots, students completed a survey about their expectations and their user experience with the different chatbots. According to Kononowicz et al. [7], employing virtual patients in education exposes students to simulated clinical cases, providing mechanisms for information gathering and clinical decision-making in a safe environment.

After using the E+DIEting_Lab platform, 79.7% of the students reported perceived improvements in practical skills related to clinical dietetics and communication had improved. Moreover, 90% acknowledged that the platform improved classroom practice, and perceived enhancement of interpersonal skills 73.9%. Self-assessed knowledge about the use of chatbots in learning improved significantly (mean rising from 2.32 ± 1.13 to 2.66 ± 1.15, *p* < 0.001), as did skills in using chatbots (from 2.39 ± 1.11 to 2.79 ± 1.11, *p* < 0.001), on a scale from 0 to 5. These findings align with prior studies indicating that chatbot-based virtual patients can enhance student’s confidence and bridge the gap between theoretical coursework and applied clinical reasoning [1,2,17].

This is consistent with the systematic review and meta-analysis conducted by Kononowicz et al. [7], who reported that, despite the limited body of evidence, virtual patients appear to be superior to traditional methods for skill development and equally effective for knowledge acquisition.

Interestingly, ratings for perceived effectiveness (3.63 ± 0.905 to 3.42 ± 1.07, *p* = 0.002) and usefulness (3.63 ± 0.920 to 3.45 ± 1.09, *p* = 0.018) declined slightly but significantly after hands-on use, despite remaining positive (>3.4/5). This pattern likely reflects refined student expectations following direct experience with the platform’s intent-based Dialogflow architecture, which unlike LLM-based systems (ChatGPT/Gemini), lacks deep contextual understanding and struggles with ambiguous/paraphrased queries [1,12,18]. Potential criticisms include limited conversational fluidity, restricted handling of non-standard questions, and absence of adaptive learning capabilities. These technical constraints mirror findings from deterministic chatbot evaluations in healthcare, where rule-based systems excel in reliability/accuracy but lag in natural interaction [5,9].

For real world applications, platforms like E+DIEting-Lab serve best as structured supplements to traditional training, providing safe, repeatable practice of standardized NCP interviews. Longitudinal follow-up should assess whether initial usability refinements translate to sustained clinical skill development, while exploring hybrid LLM-integration for enhanced conversational realism balanced against hallucinations risks.

However, ratings for the effectiveness and usefulness of the chatbot as a self-learning tool declined slightly but significantly after use (effectiveness mean 3.63 to 3.42, usefulness mean 3.63 to 3.45). Despite this, the overall perception remained positive. The main expectations at the beginning focused on acquiring practical dietetic care skills (85%) and preparing for future professional practice (75%). These slightly decreased at the end but remained high. This reflects the high expectations of students for new artificial intelligence-based tools, which many models are still unable to meet. It implies that it is necessary to continue working on improved systems that allow enhancing the interaction experience of students with chatbots. As currently configured, E+DIEting_Lab uses intent-based conversational models built on Dialogflow, which do not incorporate deep contextual or generative capacities. This design limits fluidity in conversation and restricts the chatbot’s ability to process ambiguous or paraphrased queries independently, an issue also identified in evaluations of deterministic chatbot platforms in healthcare [9].

Platforms designed to supplement classroom learning with experiential activities, such as conversational agents simulating real-world clinical scenarios, have been shown to enhance both knowledge retention and student motivation [2]. In the context of dietetics, where access to live clinical practice is often limited [3], tools such as E+DIEting_Lab provide an important bridge between theory and practice. It is worth noting that virtual patients serve as a complement to, rather than a substitute for, interaction with real patients [7].

Recent studies suggest that large language model (LLM)-based chatbots (ChatGPT, Gemini, Claude) exhibit more human-like conversational ability and stronger potential for adapting to unpredictable student interaction [12,18,19,20]. However, they present serious challenges, including concerns about academic integrity, overreliance, and AI-generated misinformation “hallucinations”.

While these more advanced systems introduce challenges in terms of safety, consistency, and regulatory oversight, their integration into future dietetic education may be studied to meet evolving user expectations and educational needs [12,20]. As highlighted by Masters [21], the integration of AI-powered chatbots in healthcare professional education, necessitates a proactive focus on ensuring data privacy, algorithmic transparency, and mitigating potential bias to uphold the ethical principles crucial for building professional competence.

Within this broader literature, the main contribution of our work lies in providing multicentre, dietetics-specific data on student perceptions of a chatbot-based virtual patient platform aligned with the NCP and IDNT, implemented across five European countries and six languages. Unlike smaller, single-institution studies with tighter experimental control and objective testing, our design prioritizes ecological validity and scalability, at the cost of internal validity. The boundaries of our evidence are therefore clear: the data speak to perceived learning gains, usability, and feasibility of E+DIEting_Lab in real teaching contexts, but cannot establish causal effects on objective knowledge, diagnostic accuracy, or clinical reasoning.

Overall, this study provides robust pilot evidence from a large multinational cohort (*n* = 475) across five European countries and six languages, featuring a dietetics-specifics chatbot platform aligned with NCP/IDNT frameworks, comprehensive GDPR-compliant technical infrastructure, and detailed pre-post evaluation capturing both perceived learning gains and usability insights.

However, several limitations should be considered when interpreting the results. The evaluation was based mainly on self-reported data, which may be subject to bias and may not fully represent actual clinical skills or knowledge acquired. The sample consisted of undergraduate students from selected European institutions, which may limit the generalizability of the findings to other educational or cultural contexts. The sample was predominantly female (75.4%, *n* = 358), which may limit generalizability to male students or other gender distribution in dietetics programs. Gender differences in technology adoption or clinical nutrition training perceptions warrant future investigation.

The response rate at follow-up was moderate, potentially introducing nonresponse bias. Some technical and linguistic differences across languages might have affected user experience uniformly. Finally, the study did not evaluate the long-term retention of knowledge or the application of skills in real clinical settings. Pre-post design isolates immediate platform effects but lacks control for semester influences.

Significant attrition (36%, 475 → 305) raises nonresponse bias concerns. Students with more favorable platform experiences may have been more motivated to complete the final survey, potentially overestimating perceived benefits. Characteristics of completers vs. non-completers were unavailable, precluding bias assessment.

Future research should include non-European institutions, postgraduate students, practicing dietitians, and culturally diverse populations to enhance global applicability. Prioritize objective outcome (quiz performance, diagnostic accuracy, clinical reasoning scores) alongside self-reported perceptions, employ control group designs, explore more diverse populations, and assess long-term skill retention to strengthen evidence for chatbot-assisted education in dietetics.

## 5. Conclusions

The implementation and pilot evaluation of the E+DIEting_Lab self-learning virtual patient chatbot platform demonstrate that structured digital simulation tools can significantly improve perceived clinical competencies. The platform, featuring multilingual, case-based virtual patient scenarios, provides students with valuable experiential learning opportunities, bridging the gap between theoretical knowledge and practical skill development. Near 80% of users reported improved practical skills and 90% acknowledged that the platform improved classroom practice. Despite these positive outcomes, challenges remain regarding conversational flexibility and meeting diverse student expectations. Lessons highlight the importance of user-centered design.

Overall, E+DIEting_Lab represents a promising direction to improve the training of future dietetic professionals with digital tools, supporting the preparation for contemporary clinical practice while underlining the need for ongoing refinement and research to maximize educational impact.

## Figures and Tables

**Table 1:** Descriptive analysis of the piloting.

	*n*	%
Initial survey completed	475	60.9
Final survey completed	305	39.1
**Sex**		
Male	117	24.6
Female	358	75.4
**Languages**		
Dutch	34	7.2
English	60	12.6
German	5	1.1
Polish	99	20.8
Portuguese	79	16.6
Spanish	198	41.7

**Table 2:** Descriptive analysis initial question.

	YES		NO	
**INITIAL QUESTION**	***n***	**%**	***n***	**%**
Have you ever used a chatbot?	206	43.4	269	56.6

**Table 3:** Descriptive analysis final questions.

	YES		NO	
**FINAL QUESTION**	***n***	**%**	***n***	**%**
Are your practical skills associated with clinical dietetics or communication updated?	243	79.7	62	20.3
Do new training tools improve practice in the dietetics classroom?	271	90	30	10
Has my capacity related to interpersonal skills increased?	224	73.9	79	26.1

**Table 4:** Descriptive and comparative analysis initial vs. final questions.

	INITIAL		FINAL		
	*n*	Mean	*n*	Mean	*p*-Value ^1^
Likert Scale 0 (Very Bad) and 5 (Very Good)					
My knowledge about the use of a chatbot in the learning process by dietetics students is?	475	2.32 ± 1.13	310	2.66 ± 1.15	<0.001
My skills in using a chatbot in the learning process as a dietetics student are?	475	2.39 ± 1.11	308	2.79 ± 1.11	<0.001
Please evaluate your knowledge and skills on how to use a chatbot as a complement—self-learning tool in your learning process.	475	2.66 ± 1.12	307	2.93 ± 1.09	<0.001
**Likert scale 0 (Not at all effective) and 5 (Highly effective)**					
What is your opinion about the effectiveness of using a chatbot as a self-learning tool for dietetics students?	475	3.63 ± 0.905	310	3.42 ± 1.07	0.002
**Likert scale 0 (Not at all useful) and 5 (Extremely useful)**					
Evaluate the usefulness of using a chatbot as a self-learning tool by dietetics students to increase competence in diagnosis and work with patient.	475	3.63 ± 0.920	309	3.45 ± 1.09	0.018

^1^ Independent Samples *t*-test.

## Data Availability

The data presented in this study are available upon request from the corresponding author. The data are not publicly available due to privacy protection.
